# The Prevalence of *Helicobacter pylori* Infection Decreases with Older Age in Atrophic Gastritis

**DOI:** 10.1155/2013/494783

**Published:** 2013-09-23

**Authors:** Shaohua Chen, Lixiong Ying, Mei Kong, Yu Zhang, Youming Li

**Affiliations:** ^1^Department of Gastroenterology, The First Affiliated Hospital, College of Medicine, Zhejiang University, No. 79 Qingchun Road, Hangzhou 310003, China; ^2^Department of Pathology, The First Affiliated Hospital, College of Medicine, Zhejiang University, Hangzhou 310003, China; ^3^Department of Oncology, Zhejiang Hospital, Hangzhou 310012, China

## Abstract

The clinical pathological characteristics of 3969 adult patients with chronic atrophic gastritis were retrospectively studied. The positivity of intestinal metaplasia and dysplasia in atrophic gastric specimens increased with age; however, *H. pylori* positivity and inflammatory activity decreased significantly with increased age. *H. pylori* infection was present in 21.01% of chronic atrophic gastritis patients, and 92.33% of the subjects with *H. pylori* infection were found to have simultaneous inflammatory activity. The intestinal metaplasia and dysplasia positivity markedly increased as the degree of gastric atrophy increased. In conclusion, the incidence of *H. pylori* infection decreased with age and correlated significantly with inflammatory activity in atrophic gastritis patients. The intestinal metaplasia and dysplasia positivity notably increased as the degree of gastric atrophy increased. Large population-based prospective studies are needed to better understand the progression of CAG.

## 1. Introduction

Chronic atrophic gastritis (CAG) is a histopathologic entity characterized by chronic inflammation of the gastric mucosa with loss of gastric glandular cells. CAG, intestinal metaplasia (IM), and epithelial dysplasia (ED) of the stomach are common and are associated with an increased risk of gastric cancer. CAG and IM are considered to be precancerous conditions. ED represents the penultimate stage of the gastric carcinogenesis sequence, defined as histologically unequivocal neoplastic epithelium without evidence of tissue invasion, and is thus a direct neoplastic precancerous lesion. ED is characterized by cellular atypia reflective of abnormal differentiation and disorganized glandular architecture.

*Helicobacter pylori* are Gram-negative bacteria that colonize the human gastric epithelium and represent one of the most common human infections worldwide*. H. pylori *infection is usually contracted in the first few years of life, and its prevalence increases with older age and lower socioeconomic status during childhood [1]. This infection is the primary inducer of CAG, IM, and ED. More than half of all humans have *H. pylori* colonies in their stomachs; however, only a minority of *H. pylori*-infected individuals develop cancer of the stomach [2]. Haziri et al. [3] reported that the prevalence of *H. pylori* infection was high in patients with CAG (66.0%), IM (71.7%), and gastric dysplasia (71.4%). In the present study, the clinical and histopathological characteristics of 3969 CAG patients from our hospital were retrospectively studied, and the relationship between *H. pylori* infection and gastric precancerous conditions was investigated. The results of this study will provide a greater understanding of CAG.

## 2. Methods

Patients with CAG diagnosed by endoscopy and histological examination from 2007 to 2012 in the First Affiliated Hospital of the College of Medicine at Zhejiang University were included in the study. One or two biopsies from the antrum were taken, and the slides were stained with hematoxylin and eosin. Cases in which *H. pylori* were identified in any of the biopsy specimens were considered positive. The presence of atrophy was assessed according to the updated Sydney System classification [4].

Fisher's exact test was used to compare the proportions of different characteristics between groups, and *P* values <0.05 were considered statistically significant.

## 3. Results

**Figure 1:**
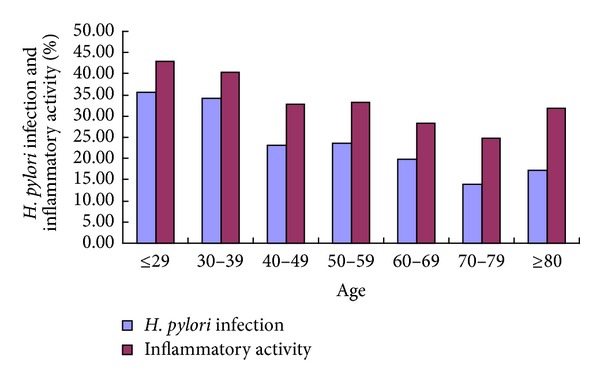
The percentage positivity of *H. pylori infection* and inflammatory activity in 3969 cases of atrophic gastritis according to age group.

There were 3969 adult patients (2051 males and 1918 females) with CAG enrolled, whose age ranged from 18 to 94 years. The distribution of the different stages of CAG, IM, and ED of the stomach according to age is shown in Table 1. In 196 cases of young adults (≤40 years), 2639 cases of middle-aged adults (41−65 years), and 1134 cases of older adults (≥66 years), the* H. pylori* infection (33.67%, 21.94%, and 16.67%), and inflammatory activity (39.8%, 31.45%, and 26.7%) decreased with age (also see in Table 1 and Figure 1). The presence of IM with young adulthood, middle and old age (80.61%, 83.86%, and 86.07%) and ED (2.04%, 3.07%, and 4.32%) in CAG patients increased with age (see also in Table 1).

### 3.1. *H. pylori* and Inflammatory Activity

There were 834 subjects (21.01%) with *H. pylori* infection; among these patients, 770 subjects (92.33%) had simultaneous inflammatory activity (Table 2). *H. pylori* positivity was 63.48% in patients with inflammatory activity, which was significantly higher than that of those without inflammatory activity (2.32%). Only 7.67% of *H. pylori*-infected patients were negative for inflammatory activity. *H. pylori* infection was significantly correlated with inflammatory activity (*P* ≤ 0.01; Table 3).

### 3.2. *H. pylori* and Precancerous Gastric Lesions

The percentage of *H. pylori* infection and inflammatory activity among 164 subjects with severe CAG was 12.80% and 23.17%, respectively, which was significantly lower than that of the mild (20.65% and 29.07%) and moderate (22.46% and 33.62%) CAG patients (Table 4). 

The *H. pylori* positivity rate in the CAG patients with IM was 20.26%, which was significantly lower than those without IM (25.08%; *P* ≤ 0.05) (Table 5). The *H. pylori* positivity was not significantly different between the CAG patients with ED and those without ED (Table 6).

### 3.3. Gastric Atrophy, Intestinal Metaplasia and Epithelial Dysplasia

IM was present in 84.33% and ED was present in 3.38% of patients with CAG. The IM and ED notably increased in positive association with more severe grade of gastric atrophy (Table 4). IM and ED appeared not to correlate with each other (Tables 5 and 6).

## 4. Discussion

*H. pylori* colonizes the stomach of more than half of the world's population, and this infection continues to play a key role in the pathogenesis of a number of gastroduodenal diseases [5]. It is hence classified as a Group A carcinogen by the World Health Organization. Epidemiological studies have determined that the attributable risk of gastric cancer conferred by *H. pylori* infection is approximately 75% [6]. Although evidence is emerging that the prevalence of *H. pylori* is declining in all age groups, the understanding of its disease spectrum continues to evolve [7].

Our study compared the *H. pylori* infection and gastric precancerous conditions in CAG by histological examination. We analyzed the presence of *H. pylori* infection in patients of different ages and found that the incidence of *H. pylori* infection decreased with age. However, several studies showed that the prevalence of *H. pylori* infection increased with age in general population in developing and developed countries [8–10], and few studies focused on *H. pylori* infection and age in atrophic patients. We also found that 92.33% of the *H. pylori*-positive patients had simultaneous inflammatory activity, which demonstrated a statistically significant relationship between *H. pylori* infection and neutrophil activation. These results are consistent with those of Khulusi et al. [11], as *H. pylori* infection could result in neutrophil activation and chronic gastritis [12]. 

Loss of normal glandular tissue is the first specific recognizable step in the precancerous cascade of gastric carcinoma [13]. Chronic *H. pylori*-induced inflammation can eventually lead to the loss of the normal gastric mucosal architecture, with destruction of the gastric glands and replacement by fibrosis and intestinal-type epithelium. This process of CAG and IM occurs in approximately half of the *H. pylori*-colonized population at sites in which inflammation is most severe [14]. The risk of CAG development depends on the distribution and pattern of chronic active inflammation.

Our study showed a low prevalence (21.01%) of *H. pylori* infection in all of the antral CAG patients, and the positivity rate decreased with growing severity of gastric atrophy. *H. pylori* infection is an established risk factor for CAG. Weck et al. reported that the odds ratio for the association between CAG and *H. pylori* infection alone was 2.9 (95% confidence interval: 2.3−3.6) [15]. What caused the low prevalence of *H. pylori* infection in the CAG patients in our study, particularly in those with severe atrophy? *H. pylori* colonization of the gastric mucosa may persist for decades or for life, unless it is eradicated by antimicrobial treatment. Perhaps the clearance of *H. pylori* infection in advanced stages of the disease is responsible for this finding. Alternatively, there is some evidence that the prevalence of these infections is declining in countries that have been rapidly developing economically, resulting from an associated improvement in the standard of living [16].

IM represents a phenotypic change from that of the normal epithelial cells of the gastric mucosa to an intestinal phenotype. It is considered to be an advanced stage of atrophy because the original glands, are replaced by metaplastic glands and chronologically, the metaplastic glands appear after the gastric glands are lost. In the present study, the majority of the CAG patients presented with IM. Furthermore, IM correlated significantly with the severity of CAG. ED is characterized by a neoplastic phenotype, both in terms of cell morphology and architectural organization. In the current study, the prevalence of ED increased with the progression of CAG. Evolution into gastric cancer was documented for all grades of dysplasia and correlated significantly with severe CAG [17].

Taken together, this study has shown that the incidence of *H. pylori* infection decreased with age and correlated significantly with inflammatory activity in CAG patients. IM and ED positivity notably increased as the degree of gastric atrophy increased. Although significant findings were revealed in the present analysis of the clinical and pathological characteristics of 3969 CAG cases, there were some limitations to our study. Only a histological examination of *H. pylori* infection was performed, which may have decreased the *H. pylori *detection rate. Further large population-based prospective studies are needed to better understand the progression of CAG.

## Figures and Tables

**Table 1:** The clinical and pathological characteristics of 3969 cases of atrophic gastritis according to age group.

Age (years)	Cases (*N*)	Gender		Gastric atrophy			Intestinal metaplasia				Dysplasia				*H. pylori *infection		Inflammatory activity	
		Male	Female	Mild	Moderate	Severe	None	Mild	Moderate	Severe	None	Mild	Moderate	Severe	Negative	Positive	Negative	Positive
≤29	28	15	13	20	5	3	8	10	6	4	27	1	0	0	18	10	16	12
30–39	129	75	54	72	52	5	20	35	55	19	128	1	0	0	85	44	77	52
40–49	605	333	272	380	212	13	104	158	270	73	585	19	1	0	465	140	407	198
50–59	1296	637	659	747	507	42	224	331	529	212	1259	32	5	0	991	305	864	432
60–69	1106	567	539	634	417	55	142	306	475	183	1067	29	8	2	887	219	794	312
70–79	689	350	339	388	269	32	107	192	271	119	657	26	3	3	593	96	519	170
≥80	116	74	42	50	52	14	17	25	46	28	112	3	1	0	96	20	79	37

Total	3969	2051	1918	2291	1514	164	622	1057	1652	638	3835	111	18	5	3135	834	2756	1213
*P *		0.134		0.000			0.048			0.468					0.000		0.000	

**Table 2:** The distribution of inflammatory (neutrophil) activity, gastric atrophy, intestinal metaplasia, and dysplasia according to the presence or absence of *H. pylori* infection.

*H. pylori* infection	Cases (*N*)	Gender		Inflammatory activity		Gastric atrophy			Intestinal metaplasia				Dysplasia			
		Male	Female	Negative	Positive	Mild	Moderate	Severe	None	Mild	Moderate	Severe	None	Mild	Moderate	Severe
Negative (%)	3135	1623 (51.77)	1512 (48.23)	2692 (85.87)	443 (14.13)	1818 (57.99)	1174 (37.45)	143 (4.56)	466 (14.87)	1988 (63.41)	564 (17.99)	117 (3.73)	3022 (96.40)	93 (2.97)	15 (0.48)	5 (0.15)
Positive (%)	834	428 (51.32)	406 (48.68)	64 (7.67)	770 (92.33)	473 (56.71)	340 (40.77)	21 (2.52)	156 (18.71)	570 (68.34)	74 (8.87)	34 (4.08)	813 (97.48)	18 (2.16)	3 (0.36)	0 (0)

Total	3969	2051	1918	2756	1213	2291	1514	164	622	2558	638	151	3835	111	18	5
*P *		0.817		0.000		0.013			0.000				0.368			

Note. The numbers in parentheses represent the percentage of inflammatory activity, gastric atrophy, intestinal metaplasia, and dysplasia in the *H. pylori-*negative and -positive groups.

**Table 3:** The distribution of *H. pylori *infection, gastric atrophy, intestinal metaplasia, and dysplasia according to the presence or absence of inflammatory activity.

Inflammatory activity	Cases (*N*)	Gender		*H. pylori *infection		Gastric atrophy			Intestinal metaplasia				Dysplasia			
		Male	Female	Negative	Positive	Mild	Moderate	Severe	None	Mild	Moderate	Severe	None	Mild	Moderate	Severe
Negative (%)	2756	1399 (50.76)	1357 (49.24)	2692 (97.68)	64 (2.32)	1625 (58.96)	1005 (36.47)	126 (4.57)	414 (15.02)	1763 (63.97)	476 (17.27)	103 (3.74)	2655 (96.34)	85 (3.08)	12 (0.44)	4 (0.14)
Positive (%)	1213	652 (53.75)	561 (46.25)	443 (36.52)	770 (63.48)	666 (54.91)	509 (41.96)	38 (3.13)	208 (17.15)	795 (65.54)	162 (13.36)	48 (3.95)	1180 (97.28)	26 (2.14)	6 (0.50)	1 (0.08)

Total	3969	2051	1918	3135	834	2291	1514	164	622	2558	638	151	3835	111	18	5
*P *		0.083		0.000		0.001			0.020				0.381			

Note. The numbers in parentheses represent the percentage of *H. pylori infection*, gastric atrophy, intestinal metaplasia, and dysplasia in the inflammatory activity-negative and -positive groups.

**Table 4:** The distribution of *H. pylori* infection, inflammatory (neutrophil) activity, intestinal metaplasia and dysplasia according to the grade of gastric atrophy.

Gastric atrophy	Cases (*N*)	Gender		*H. pylori* infection		Inflammatory activity		Intestinal metaplasia		Dysplasia	
		Male	Female	Negative	Positive	Negative	Positive	Negative	Positive	Negative	Positive
Mild (%)	2291	1144	1147	1818	473 (20.65)	1625	666 (29.07)	496	1795 (78.35)	2230	61 (2.66)
Moderate (%)	1514	821	693	1174	340 (22.46)	1005	509 (33.62)	117	1397 (92.27)	1453	61 (4.03)
Severe (%)	164	86	78	143	21 (12.80)	126	38 (23.17)	9	155 (94.51)	152	12 (7.32)

Total (%)	3969	2051	1918	3135	834 (21.01)	2756	1213 (30.56)	622	3347 (84.33)	3835	134 (3.38)
*P *		0.034		0.013		0.001		0.000		0.000	

Note. The numbers in parentheses represent the percentage of *H. pylori* infection, inflammatory activity, intestinal metaplasia and dysplasia in patients with different degrees of gastric atrophy.

**Table 5:** The distribution of *H. pylori* infection, inflammatory (neutrophil) activity, gastric atrophy, and dysplasia according to the presence or absence of intestinal metaplasia.

Intestinal metaplasia	Cases (*N*)	Gender		*H. pylori* infection		Inflammatory activity		Gastric atrophy			Dysplasia	
		Male	Female	Negative	Positive	Negative	Positive	Mild	Moderate	Severe	Negative	Positive
Negative (%)	622	271	351	466	156 (25.08)	414	208 (33.44)	496 (79.74)	117 (18.81)	9 (1.45)	606	16 (2.57)
Positive (%)	3347	1780	1567	2669	678 (20.26)	2342	1005 (30.03)	1795 (53.63)	1397 (41.74)	155 (4.63)	3229	118 (3.53)

Total (%)	3969	2051	1918	3135	834 (21.01)	2756	1213 (30.56)	2291 (57.72)	1514 (38.15)	164 (4.13)	3835	134 (3.38)
*P *		0.000		0.000		0.02		0.000			0.513	

Note. The numbers in parentheses represent the percentage of *H. pylori* infection, inflammatory activity, gastric atrophy, and dysplasia in the intestinal metaplasia-negative and -positive groups.

**Table 6:** The distribution of *H. pylori* infection, inflammatory (neutrophil) activity, gastric atrophy and intestinal metaplasia according to the presence or absence of dysplasia.

Dysplasia	Cases (*N*)	Gender		*H. pylori* infection		Inflammatory activity		Gastric atrophy			Intestinal metaplasia	
		Male	Female	Negative	Positive	Negative	Positive	Mild	Moderate	Severe	Negative	Positive
Negative (%)	3835	1966	1869	3022	813 (21.20)	2655	1180 (30.77)	2230 (58.15)	1453 (37.89)	152 (3.96)	606	3229 (84.2)
Positive (%)	134	85	49	113	21 (15.67)	101	33 (24.63)	61 (45.52)	61 (45.52)	12 (8.96)	16	118 (88.06)

Total (%)	3969	2051	1918	3135	834 (21.01)	2756	1213 (30.56)	2291 (57.72)	1514 (38.15)	164 (4.13)	622	3347 (84.33)
*P *		0.031		0.368		0.381		0.000			0.513	

Note. The numbers in parentheses represent the percentage of *H. pylori* infection, inflammatory activity, gastric atrophy and intestinal metaplasia in the dysplasia-negative and -positive groups.

## Abbreviations

*H. pylori*: *Helicobacter pylori*
CAG: Chronic atrophic gastritis
IM: Intestinal metaplasia
ED: Epithelial dysplasia.
